# New Magnetic Resonance Imaging Index for Renal Fibrosis Assessment: A Comparison between Diffusion-Weighted Imaging and T1 Mapping with Histological Validation

**DOI:** 10.1038/srep30088

**Published:** 2016-07-21

**Authors:** I. Friedli, L. A. Crowe, L. Berchtold, S. Moll, K. Hadaya, T. de Perrot, C. Vesin, P.-Y. Martin, S. de Seigneux, J.-P. Vallée

**Affiliations:** 1 Division of Radiology, Department of Radiology and Medical Informatics Geneva University Hospitals and Faculty of Medicine of the University of Geneva, Switzerland; 2 Service of Nephrology, Department of Internal Medicine Specialties, Geneva University Hospitals, University of Geneva, Faculty of Medicine, Geneva, Switzerland; 3 Division of Pathology, Geneva University Hospitals and Faculty of Medicine of the University of Geneva, Switzerland; 4 Divisions of Nephrology and Transplantation, Geneva University Hospitals and Faculty of Medicine of the University of Geneva, Switzerland; 5 Division of Cell Physiology and Metabolism, Geneva University Hospitals and Faculty of Medicine of the University of Geneva, Switzerland

## Abstract

A need exists to noninvasively assess renal interstitial fibrosis, a common process to all kidney diseases and predictive of renal prognosis. In this translational study, Magnetic Resonance Imaging (MRI) T1 mapping and a new segmented Diffusion-Weighted Imaging (DWI) technique, for Apparent Diffusion Coefficient (ADC), were first compared to renal fibrosis in two well-controlled animal models to assess detection limits. Validation against biopsy was then performed in 33 kidney allograft recipients (KARs). Predictive MRI indices, ΔT1 and ΔADC (defined as the cortico-medullary differences), were compared to histology. In rats, both T1 and ADC correlated well with fibrosis and inflammation showing a difference between normal and diseased kidneys. In KARs, MRI indices were not sensitive to interstitial inflammation. By contrast, ΔADC outperformed ΔT1 with a stronger negative correlation to fibrosis (R^2^ = 0.64 against R^2^ = 0.29 p < 0.001). ΔADC tends to negative values in KARs harboring cortical fibrosis of more than 40%. Using a discriminant analysis method, the ΔADC, as a marker to detect such level of fibrosis or higher, led to a specificity and sensitivity of 100% and 71%, respectively. This new index has potential for noninvasive assessment of fibrosis in the clinical setting.

Kidney Interstitial Fibrosis (IF) is defined as the abnormal deposition of collagen and related proteins in the cortical renal interstitium. IF is a common histological abnormality present in all types of renal disease and is considered to be crucial for the prediction of functional recovery of the kidney and prognosis in most renal diseases^1^. In kidney allograft recipients (KARs), IF determines allograft prognosis and is used to adapt treatment^2^,^3^,^4^,^5^,^6^,^7^,^8^. IF is currently evaluated by histological analysis of kidney biopsies, which may be complicated by serious bleeding^9^,^10^. In addition, these random biopsies are subject to sampling bias and are difficult to perform repeatedly due to potential complications. Finally, there is ongoing debate over the best method to estimate IF histologically, in a reproducible manner, in KARs and chronic kidney diseases (CKD) patients^11^,^12^,^13^. Diagnostic tools and noninvasive biomarkers for the detection of IF are essential to complement serologic markers and biopsies in order to improve the prognostic and follow-up of KARs, and CKD patients in general. Noninvasive methods such as elastography and fibroscan have been validated for the fibrosis assessment of organs such as the liver^14^,^15^. However, there exists currently no recognized noninvasive method for fibrosis quantification in the kidney. Several Magnetic Resonance Imaging (MRI) approaches are emerging to measure fibrosis, including T1 mapping and Diffusion-Weighted MRI (DWI) as the two most promising methods^16^,^17^. The first MRI method, T1 mapping, is a parametric map where each pixel of a kidney image represent the T1 spin-lattice relaxation time. T1 relaxation is tissue specific, depending on the molecular environment of the water molecules. In addition to tissue composition, T1 is sensitive to pathological changes occurring in the tissues. Increased cardiac T1 has been shown to be very efficient to detect diffuse myocardial fibrosis^18^ and the use of T1 mapping for the diagnosis and monitoring of cardiomyopathies is currently the subject of intense research. In normal renal parenchyma, T1 mapping can differentiate cortex and medulla^19^. Less is known about the relationship between T1 and renal cortical IF. In transplanted mice, T1 was increased in allograft kidneys exhibiting marked IF^20^. As macrophage and T-lymphocyte infiltration was also present in the rejecting kidneys, the exact relationship between T1 and IF could not be inferred from this study. In patients, cortical T1 was negatively correlated with renal function for native and transplanted kidneys suggesting that T1 could also be used to assess kidney IF^21^,^22^. However, no histological assessment of IF was available for validation of these clinical studies. The second promising MRI method to assess IF is DWI, which is sensitive to the Brownian motion of water molecules in tissue and is most often quantified using the Apparent Diffusion Coefficient (ADC). ADC is routinely used as the best marker of cerebral ischemia^23^ and is emerging for kidney diseases. Renal ADC was decreased in mice with increased cellular density or interstitial remodeling, as in IF^24^, and in an acute kidney injury model compared to control mice^25^. Studies using dedicated small animal high field systems (e.g. 7T), with gradient strength and field homogeneity much superior to clinical MR scanners, have proven the efficacy of ADC for monitoring progression of IF in mice with unilateral ureteral obstruction (UUO)^26^. In CKD patients, a decrease of ADC significantly correlated with the increase of IF obtained by histopathology^27^,^28^,^29^. Recently Zhao *et al*. observed, in native kidney diseases, a correlation between cortical or medullary ADC values and IF, as assessed by histology^29^. However, this interesting result remains to be confirmed by other studies as kidney DWI is extremely challenging due to low image resolution and artifacts, including respiratory motion and image distortion^30^. Absolute renal ADC values show wide inter-individual variation^31^ and the absence of consensus regarding reference values for normal renal ADC precludes routine clinical use. Before large-scale clinical adoption, more robust DWI sequences need to be developed and validated. Recently, RESOLVE, a new DWI sequence with segmented acquisition, gave enhanced image quality in volunteers with normal renal function by reducing image distortion and improving the differentiation of ADC between cortex and medulla^32^. RESOLVE has improved the diagnostic performance of DWI in breast, head and pelvis examinations^33^,^34^,^35^,^36^ and similar benefit could be expected in renal patients. However, the performance of RESOLVE for IF assessment in patients has not yet been evaluated. Our goal was, therefore, to compare the performance of Modified Look-Locker Inversion-recovery (MOLLI) T1 mapping and RESOLVE DWI to assess renal IF. First, T1 mapping and RESOLVE DWI protocols were adapted to scan rat models on a clinical 3T MRI scanner. This experimental step was important to evaluate the sensitivity of the MR parameters to detect low levels of fibrosis in well-controlled animal models. These protocols were then applied to KARs undergoing planned biopsy. IF is indeed an important endpoint for adaption of therapy decisions in this population, as well as a marker for allograft prognosis.

## Results

### Unilateral ureteral obstruction in rats induced severe interstitial fibrosis, which was detected by T1 mapping and DWI

**Figure 1:**
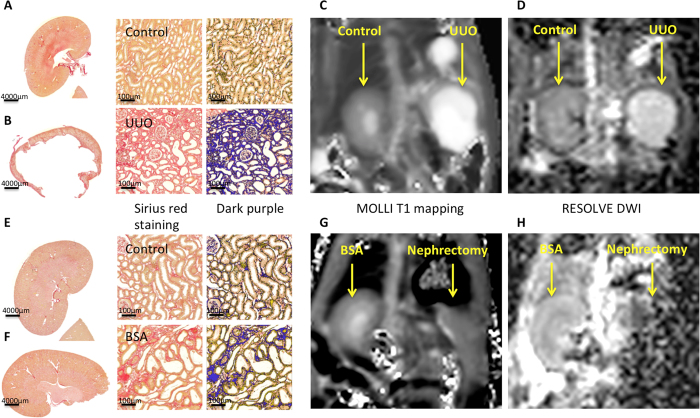
Representative histological and MR images of the unilateral ureteral obstruction (UUO) model at 2 weeks (**A–D**) and bovine serum albumin (BSA) nephritis model (**E–H**). Macroscopy of the contralateral normal (**A**) and obstructed kidneys (**B**) in the UUO model and the BSA (**F**) and sham kidneys (**E**) was followed by the zoomed Sirius red staining, and its threshold quantified in dark purple, showing severe fibrosis in the UUO model and moderate bands of fibrosis in the BSA model. Good quality coronal MRI images of MOLLI T1 maps (**C,G**) and RESOLVE ADC maps (**D,H**) were obtained for both the UUO and BSA models.

**Figure 2:**
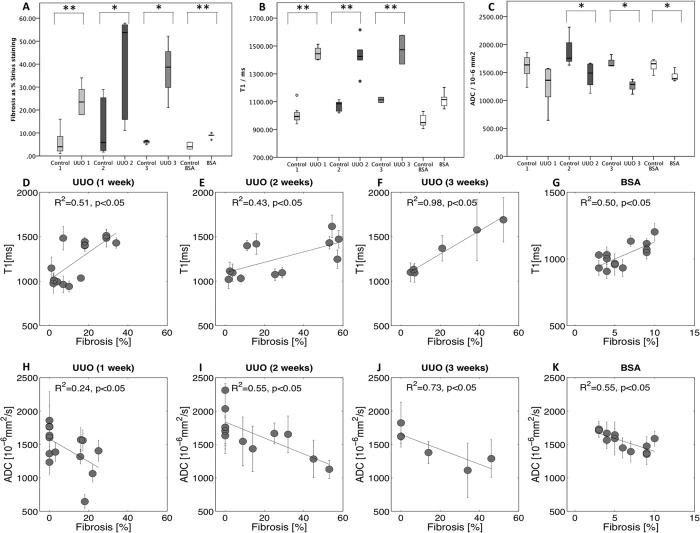
Histological and MRI results box plot for the UUO and BSA model. The 3 boxes plot illustrate the differences between the control and the model for histological results (**A**), the mean T1 [ms] (**B**) and mean ADC [10^−6^ mm^2^/s] (C) for UUO and BSA: UUO at time point 1 week (UUO1), 2 weeks (UUO2), 3 weeks (UUO3) and BSA at time point 3 weeks. In UUO, the contralateral kidney served as control. Data were obtained in 29 rats (7 for UUO1, 6 for UUO2, 3 for UUO3, 5 in the BSA group and 8 controls), with p < 0.001 (**) and with p < 0.05 (*). (**B**) A highly significant difference in T1 was revealed between the control and obstructed kidneys in the UUO model at the three time points but only a trend was observed for the BSA model (p = 0.06). In all case, T1 strongly correlated with the percentage of cortical IF as assessed by Sirius red staining (R^2^ = 0.51 at 1 week (**D**), R^2^ = 0.43 at 2 weeks (**E**), R^2^ = 0.98 at 3 weeks (**F**), p < 0.05) and R^2^ = 0.50, p < 0.05 for the BSA 3 weeks (**G**). ADC was significantly different between the control and both the UUO model at 2 and 3 weeks (p = 0.013 and p = 0.014) and the BSA model (p = 0.007). The difference in ADC was not significant in the mild UUO model at time point 1 week (p = 0.052) (**C**). In all cases, ADC inversely correlated with the percentage of cortical IF as assessed by Sirius red staining (R^2^ = 0.24 at 1 week (**H**), R^2^ = 0.55 at 2 weeks (**I**), R^2^ = 0.73 at 3 weeks (**J**), p < 0.05) and R^2^ = 0.55, p < 0.05 for the BSA 3 weeks (**K**).

Significant difference was found between contralateral (Fig. 1A) and obstructed kidneys (Fig. 1B) in the UUO rat model. As expected, obstructed kidneys displayed tubular dilatation, moderate to severe fibrosis, interstitial inflammation and tubular atrophy as shown in the histological section of a UUO at 2 weeks after animal surgery (Fig. 1B). Kidney cortical fibrosis was quantified by unpolarized Sirius red staining at 1 (n = 7), 2 (n = 6) and 3 (n = 3) weeks and was compared to the non-obstructed, contralateral kidney. Quantification of unpolarized Sirius red staining was significantly higher in the obstructed kidneys compared to the contralateral cortex at all 3 time points (p < 0.05) (Fig. 2A).

**Figure 3:**
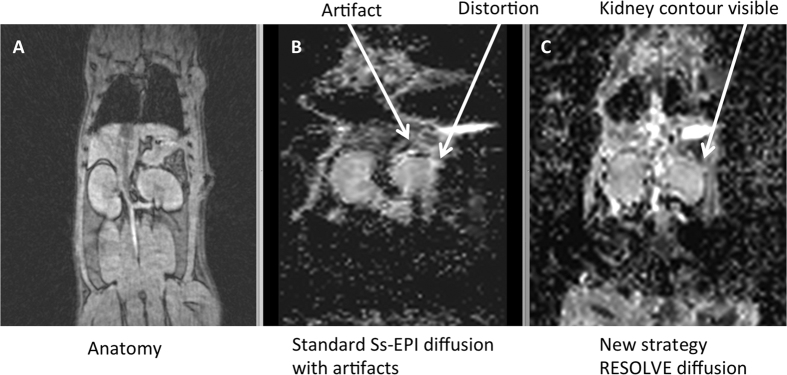
Comparison between single-shot (ss-EPI) and RESOLVE DWI MR sequences in a small animal. Both DWI images were compared to GRE anatomical MR images (**A**). Standard ss-EPI MR sequences showed severe distortion at the kidney edges (**B**). In 14% of kidneys, for the ss-EPI images, the parenchyma completely disappeared due to distortions. RESOLVE MR sequences (**C**) considerably reduced artifact, enabling therefore analysis.

**Figure 4:**
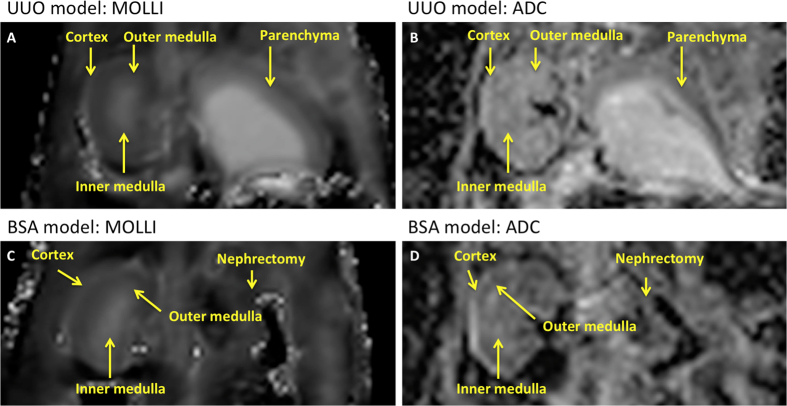
Representative T1 maps and ADC maps of the unilateral ureteral obstruction (UUO) model (**A,B**) and bovine serum albumin (BSA) nephritis model (**C,D**). First column, coronal MOLLI T1 maps in the UUO model (**A**) and in the BSA example (**C**) followed by coronal ADC map obtained with RESOLVE sequence (**B**,**D**). The renal cortex, and the outer and inner medulla were identified on the BSA model and sham, as well as the contralateral unobstructed kidney of the UUO rats. Layers were not distinguished on the left obstructed UUO kidney due to renal parenchyma atrophy.

In a pilot study to optimize the MRI protocol, traditional single-shot DWI (ss-EPI) images were not suitable for ADC analysis in 14% of whole rat kidneys, compared to images obtained with RESOLVE MRI sequence. Figure 3B shows a typical example of the severe distortion present on a standard ss-EPI image of rat kidney. The parenchyma completely disappeared due to susceptibility artifact and related distortions. On the contrary, RESOLVE DWI improved image quality by reducing distortions enabling analysis of all kidneys (Fig. 3C). Images were therefore acquired with RESOLVE for the comparison with T1 and histopathology. Figures 1C and 4A show good quality T1 maps obtained in all the animals except one, which due to a technical problem during acquisition was excluded from the T1 evaluation. Obstructed and contralateral kidneys were clearly identified by MRI with both T1 mapping (Figs 1C and 4A) and RESOLVE (Figs 1D and 4B). The cortical and medullary layers could be visualized separately on T1 mapping and RESOLVE in the contralateral, but not in the obstructed kidney as a result of the renal parenchyma atrophy observed in the obstructed kidney (Fig. 1B). Regarding T1 quantification, the T1 value was significantly higher in the obstructed kidney compared to the contralateral kidney at the three time points (p < 0.05) (Fig. 2B). When considering all animals, T1 consistently increased with the percentage of cortical IF as assessed by Sirius red staining with a significant correlation at all 3 time points (R^2^ = 0.51 at 1 week (Fig. 2D), R^2^ = 0.43 at 2 weeks (Fig. 2E) and R^2^ = 0.98 at 3 weeks (Fig. 2F), p < 0.05 T1 was also significantly correlated with interstitial inflammation at 2 weeks (R^2^ = 0.30, p = 0.054 at 1 week, R^2^ = 0.76, p < 0.05 at 2 weeks and R^2^ = 0.76, p = 0.053 at 3 weeks). Measured from DWI, the ADC was significantly lower in the obstructed kidney than in the contralateral kidney at 1 (p = 0.05), 2 and 3 weeks (p < 0.05) (Fig. 2C). ADC decreased with increasing IF (R^2^ = 0.24 at 1 week (Fig. 2H), R^2^ = 0.55 at 2 weeks (Fig. 2I) and R^2^ = 0.73 at 3 weeks (Fig. 2J), p < 0.05) and interstitial inflammation at 2 and 3 weeks (R^2^ = 0.22, p = 0.089 at 1 week, R^2^ = 0.55, at 2 weeks and R^2^ = 0.90 at 3 weeks, p < 0.05). In summary, MRI sequences with T1 mapping and ADC obtained from DWI were both able to differentiate parenchyma of obstructed kidney from the contralateral control in the UUO model.

### Immunologic nephritis induced moderate kidney IF, which was detected by T1 and ADC MRI

To further evaluate the sensitivity of our imaging protocols in a model of milder renal IF, immunologic nephritis was induced by repeated injections of bovine serum albumin (BSA)^37^. On histology, by unpolarized Sirius red staining, moderate bands of cortical fibrosis with modest foci of interstitial inflammation were present in BSA kidneys (n = 5), compared to sham animals (n = 8) (p < 0.05) as shown in representative histological images (Fig. 1E,F). T1 mapping and DWI of good quality were obtained in all the animals. The different layers of parenchyma were identified by both T1 mapping (shown in Figs 1G and 4C), and the RESOLVE sequence (shown in Figs 1H and 4D). Cortical T1 values showed a trend to be higher in the BSA group compared to the sham group (p = 0.06) (Fig. 2B). The BSA group showed also, a strong positive correlation between cortical T1 and IF (R^2^ = 0.50, p < 0.05) (Fig. 2G). However, when considering only the inflammation score and T1 values, no correlation was found in this population (R^2^ = 0.017, p = 0.76).

Regarding the DWI, the cortical ADC decreased significantly in the BSA group compared to the sham group (p < 0.05) and a strong negative correlation was recorded with increasing IF (R^2^ = 0.55, p < 0.05) (Fig. 2K). In this model also, cortical T1 and ADC performed similarly to detect IF with a significant decrease of the cortical ADC and a significant increase of cortical T1.

### In kidney allograft recipients, ADC values showed a stronger correlation than T1 to IF

**Figure 5:**
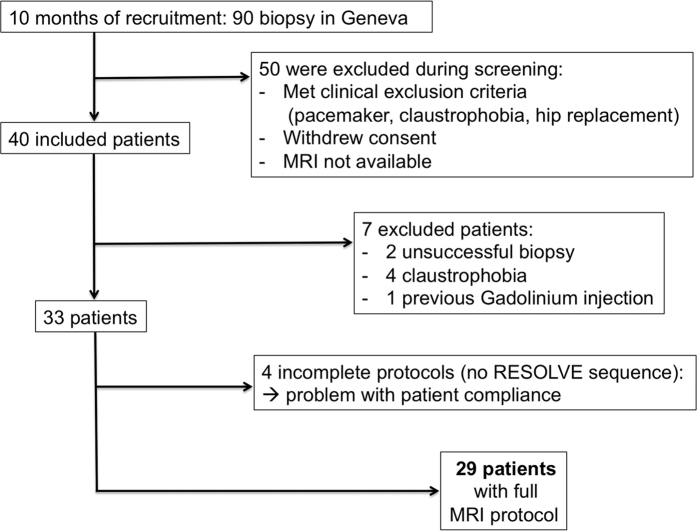
Flowchart illustrating patient recruitment.

After validation of our MRI protocol to detect IF in rats using the clinical 3T MR, the same MR protocol was translated with appropriately adjusted resolution and field of view parameters to KARs undergoing kidney biopsy. In 4 patients, only T1 values were acquired due to problems with patient compliance as detailed in the flowchart illustrating patient recruitment (Fig. 5).

**Figure 6:**
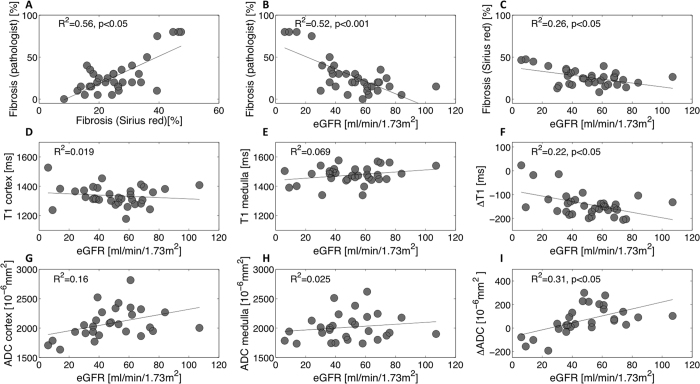
eGFR versus Fibrosis, T1 and ADC in kidney allograft recipients undergoing routine kidney biopsy (n = 32, 28 and 32). eGFR was calculated using the CKD-EPI equation, except in one patient presenting with AKI at the time of biopsy. A strong positive correlation between IF estimated by pathologist-assessed Masson trichrome and IF quantified by Sirius red staining was measured (R^2^ = 0.56, p < 0.05) (**A**). Negative correlations were measured between IF (Masson trichrome) and eGFR (R^2^ = 0.52, p < 0.001) (**B**) and between IF (Sirius red) and eGFR (R^2^ = 0.26, p = 0.002) (**C**). T1 (cortex, medulla) and eGFR were non-correlated (R^2^ = 0.019 in the cortex (**D**) and R^2^ = 0.069 in the medulla (**E**)). However, the cortico-medullary difference ΔT1 showed a negative tendency with the increase of eGFR (**F**). Compared to cortex or medulla alone (**G**,**H**), ΔADC also improved the correlation with eGFR (R^2^ = 0.31, p < 0.05) (**I**).

**Figure 7:**
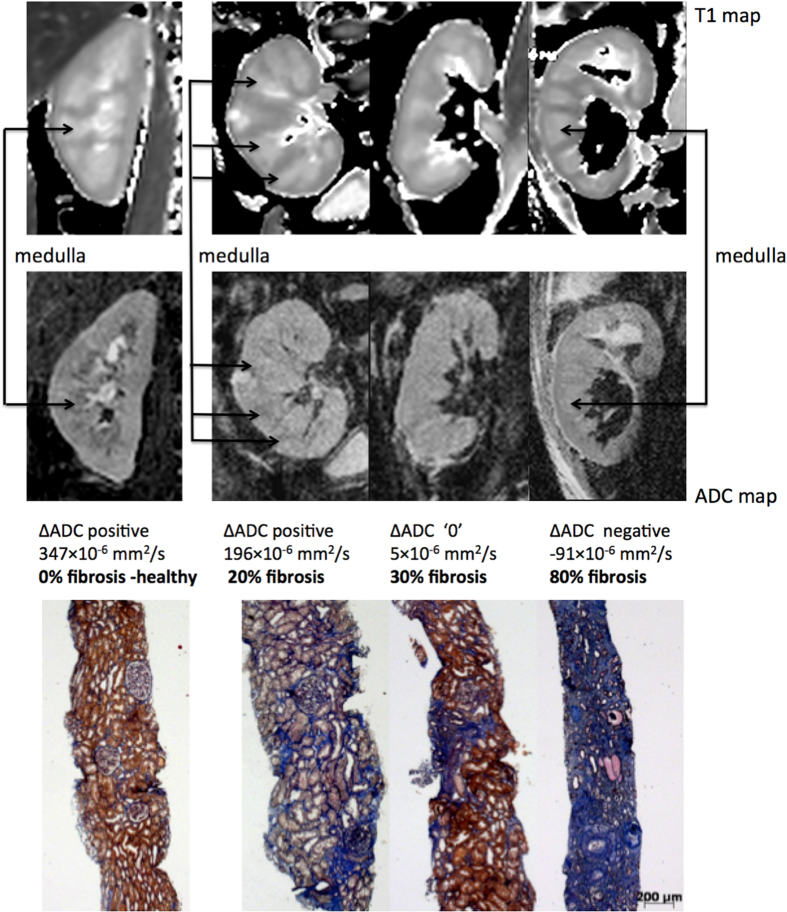
Representative biopsy and MR images patients. Morphological MOLLI T1 map used for the positioning of the regions of interest (top row) and ADC maps (lower row) for 3 patients showing the different ΔADC cases: positive, zero and negative; along with the corresponding fibrosis levels from histology (Masson trichrome staining).

As gold standard, cortical IF was assessed by automatic unpolarized Sirius red quantification of the biopsied cortex^3^,^38^ and also, by classical visual estimation by an experienced pathologist, using Masson trichrome staining. Although the latter is the method used in clinical routine, both methods were investigated in this study and a strong positive correlation between pathologist-assessed Masson trichrome and unpolarized Sirius red quantification for IF assessment was measured (R^2^ = 0.56, p < 0.05) (Fig. 6A). Strong negative correlations were also measured between eGFR^39^ and IF assessed by Masson trichrome (R^2^ = 0.52, p < 0.001) (Fig. 6B) and by Sirius red (R^2^ = 0.26, p < 0.05) (Fig. 6C). Except in 3 patients with a high level of IF, T1 maps demonstrated a clear cortico-medullary difference as shown in the first row of Fig. 7. The range of T1 values was 1175 to 1527 ms for the cortex and 1327 to 1576 ms for the medulla. T1 was not correlated with eGFR (R^2^ = 0.019 in the cortex (Fig. 6D) and R^2^ = 0.069 in the medulla (Fig. 6E)). To decrease inter-individual variability we calculated the cortico-medullary difference for T1 values (ΔT1), which ranged from −206 to 23 ms. ΔT1 showed a positive correlation with eGFR (R^2^ = 0.22, p < 0.05) (Fig. 6F).

**Figure 8:**
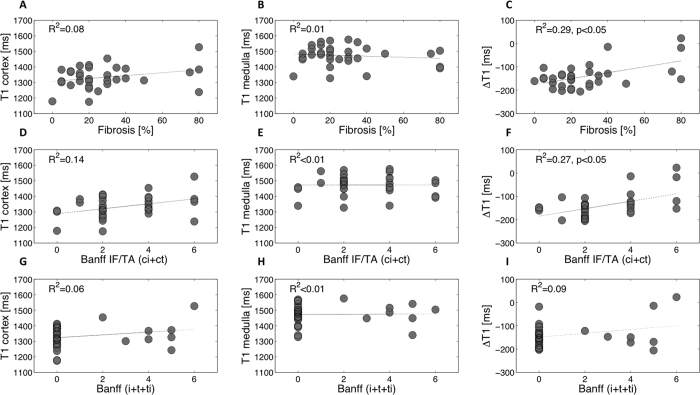
Correlations between histopathological results (fibrosis estimated by pathological assessment of Masson trichrome (**A–C**), Banff IF/TA (ci+ct) (**D–F**) and Banff (i+t+ti) (**G–I**)) and T1 values in the cortex and medulla, and ΔT1 in 33 KARs. ΔT1 (in ms) was calculated as the difference between cortical and medullary T1. In all case, no correlation was found when comparing T1 to histopathological results in the cortex and medulla alone. A moderate correlation was found between ΔT1 and the percentage of cortical IF estimated by pathological assessment of Masson trichrome (**C**) and also, between ΔT1 and fibrosis estimated by Banff IF/TA (ci+ct) with respectively (R^2^ = 0.29 and R^2^ = 0.27, p < 0.05) (**F**).

No correlation was found between absolute T1 values and IF as assessed either from Masson trichrome (R^2^ = 0.087 in the cortex (Fig. 8A) and R^2^ = 0.012 in the medulla (Fig. 8B)) or from Sirius red (R^2^ = 0.18 in the cortex and R^2^ = 0.016 in the medulla) whereas ΔT1 and IF showed moderate correlations (R^2^ = 0.29, p < 0.05 from Masson trichrome (Fig. 8C) and R^2^ = 0.18, p < 0.05 from Sirius red). A significant but moderate correlation was measured between the Banff scoring system for chronic interstitial lesions (interstitial fibrosis and tubular atrophy, ci+ct) and ΔT1 with R^2^ = 0.27, p = 0.002 (Fig. 8F) but not with the T1 values in either the cortex or medulla alone (R^2^ = 0.13, R^2^ < 0.01 respectively (Fig. 8D,E)). Similarly, no correlation was measured when comparing the T1 or ΔT1 and the inflammation as assessed by adding three variables of the Banff pathology score^40^,^41^ representing tubulo-interstitial inflammation (i+t+ti) (R^2^ = 0.06 in the cortex (Fig. 8G), R^2^ < 0.01 in the medulla (Fig. 8H) and R^2^ = 0.09 with the ΔT1 (Fig. 8I)).

**Figure 9:**
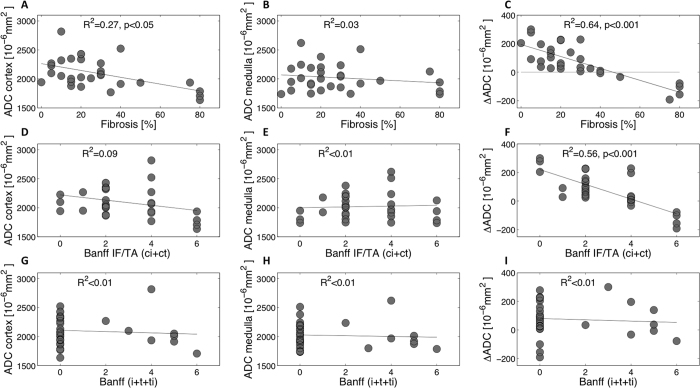
Correlations between histopathological results (fibrosis estimated by pathological assessment of Masson trichrome (**A–C**), Banff IF/TA (ci+ct) (**D–F**) and Banff (i+t+ti) (**G–I**)) and ADC values in the cortex and medulla, and ΔADC of 29 KARs. ΔADC (in 10^−6^ mm^2^/s) was calculated as the difference between cortical and medullary ADC. Cortical IF (estimated by pathological assessment of Masson trichrome) was moderately correlated with cortical ADC (**A**) but strongly with ΔADC (R^2^ = 0.64, p < 0.001) (**C**). All patients with more than 40% IF presented a negative ΔADC. A strong negative correlation was also measured with Banff IF/TA (ci+ct), whereas no correlation with interstitial inflammation assessed by Banff (i+t+ti) was measured (**G–I**).

**Figure 10:**
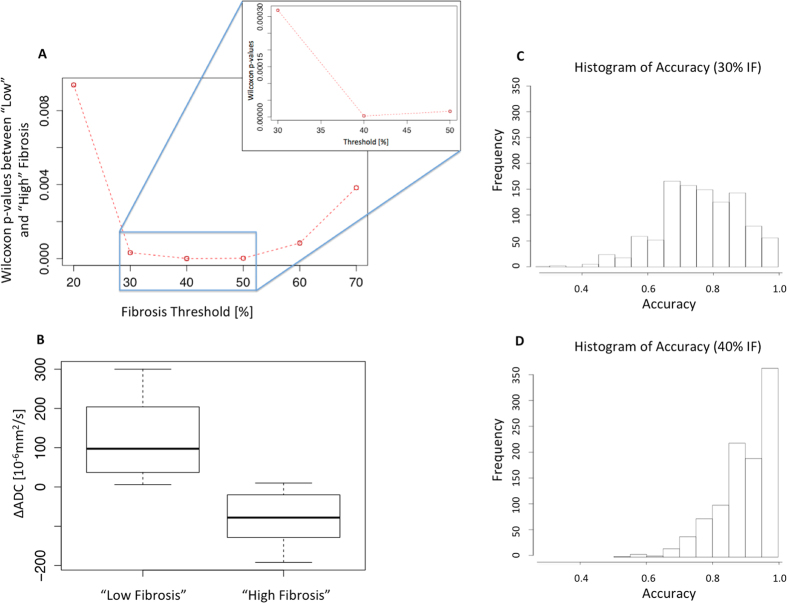
Evaluation of the limit at 40% IF for the definition of “Low Fibrosis” versus “High Fibrosis” detectable using the ΔADC index. The percentage of IF was defined as binary factor using 2 groups: ‘Low Fibrosis’ and ‘High Fibrosis’. (**A**) Wilcoxon p-values between ‘Low Fibrosis’ and ‘High Fibrosis’ groups were computed for IF thresholds between 10% and 70% by increment of 10% (with zoom shown for 30% to 50%). The best separation between groups “Low Fibrosis” and “High Fibrosis” was found at a limit of 40% with the lowest p-value computed (p = 2.6 × 10^−6^). The other separating limits were 10% (p = 2.0 × 10^−2^), 20% (p = 9.4 × 10^−3^), 30% (p = 3.2 × 10^−4^), 40% (p = 2.6 × 10^−6^), 50%(p = 1.7 × 10^−5^), 60% (p = 8.4 × 10^−5^), 70% (p = 3.4 × 10^−3^). Due to the large p-value the 10% threshold is not included on the plot to keep the vertical scale of the remaining points visible. (**B**) Classification of each ΔADC with this limit at 40% into separate groups as ‘Low Fibrosis’ and ‘High Fibrosis’ groups. At this level of IF, KARs with positive ΔADC and KARs with negative ΔADC can be separated without overlap between the interquartile range (boxes). (**C**,**D**) The accuracy of the limit of 40% IF to separate ‘Low Fibrosis’ to ‘High Fibrosis’ groups according to the ΔADC was 91% with 95% CI [0.77–0.99]. Bootstrap values were shifted close to 1.0 at a level of 40% (**D**) compared to the accuracy distribution at 30% (**C**), indicating that 40% IF was more accurate to separate “Low” to “High” fibrosis.

High image quality was obtained by the RESOLVE sequence, with only few susceptibility artifacts at the edge of the parenchyma (Fig. 7, 2^nd^ row). The ADC values [x10^−6^mm^2^/s] had a large range from 1634 to 2816 for the cortex and from 1735 to 2620 for the medulla. ADC images demonstrated 3 different contrast combinations: ADC lower in the cortex than medulla (as shown in the healthy kidney and the KAR with 20% IF in Fig. 7), no ADC difference between the cortex and the medulla (as shown in the KAR with 30% IF), and higher ADC in the cortex than the medulla (as shown in the KAR with 80% IF). A moderate negative correlation was found between absolute cortical ADC and IF assessed by Masson trichrome (R^2^ = 0.27, p < 0.05) (Fig. 9A) but not by Sirius red (R^2^ = 0.025). Cortical ADC and eGFR were not correlated (R^2^ = 0.16) (Fig. 6G). Medullary ADC was also not correlated with eGFR (R^2^ = 0.025 (Fig. 6H)) nor with cortical IF (R^2^ = 0.03 by Masson trichrome (Fig. 9B), R^2^ = 0.02 from Sirius red)). Given large inter-individual variation, we derived the index of the difference between cortical and medullary ADC (ΔADC), which ranged from −193 to 300 (x10^−6^mm^2^/s). The ΔADC index improved significantly the correlation with eGFR (R^2^ = 0.31, p < 0.05 (Fig. 6I)), as well as with IF (R^2^ = 0.64, p < 0.05 by Masson trichrome (Fig. 9C) and R^2^ = 0.37, p < 0.05 by Sirius red). In addition to this strong correlation, a negative ΔADC was observed in all patients with more than 40% IF (Fig. 9C). A strong correlation was measured between Banff chronic interstitial lesion gradations for interstitial fibrosis and tubular atrophy (ci and ct) and ΔADC with R^2^ = 0.56, p < 0.001 (Fig. 9F) but not with either the cortex or medulla ADC alone (R^2^ = 0.09, R^2^ < 0.01 respectively) (Fig. 9D,E). No correlation was measured when comparing the ADC and the inflammation scoring in the tubulo-interstitium measured by Banff (i+t+ti) (R^2^ < 0.01 cortex, medulla and ΔADC (Fig. 9G–I)). Based on R^2^ correlation comparison using a Fisher Z-transform test, ΔADC outperformed ΔT1 in assessment of IF assessed by Masson trichrome and by Banff IF/TA (ci+ct) (p < 0.001). Correlation coefficients between ΔADC and IF assessed by Masson trichrome and between ΔADC and Banff IF/TA (ci+ct) were not statistically different (p = 0.641). We further concentrated on the ΔADC to validate a limit of detection for IF with nonparametric Wilcoxon and Bootstrap methods. In the first analysis, by sequentially separating the population into 2 groups: ‘High IF’ and ‘Low IF’ with different possible thresholds, Wilcoxon p-values of all the possible thresholds were computed and the lowest p-value was found for a threshold of 40% (p = 2.6 × 10^−6^ (Fig. 10)). By using this level to define KARs as having fibrotic disease or not, and discriminant linear analysis, ΔADC as predictive index provided a sensitivity and specificity of 71 and 100% respectively. Applying the bootstrap method, the accuracy was estimated at 91% with 95% CI [0.77–0.99].

Strong reproducibility of ADC and T1 measurement in the cortex and medulla was found between two readers. For each patient independently, all ICC were superior to 0.91 [95% CI:0.92–0.99] for ADC cortex, ADC medulla and ΔADC and ICC>0.90 [95% CI:0.63–0.97] for T1 cortex, T1 medulla and ΔT1. Correlation coefficients between the two readers were R^2^ = 0.96 for the ADC evaluation in the cortex, R^2^ = 0.97 in the medulla and R^2^ = 0.95 for the ΔADC (p < 0.05). For T1, correlation coefficients between the two readers were R^2^ = 0.737 (p = 0.001) for the cortex, R^2^ = 0.696 (p = 0.03) for the medulla and R^2^ = 0.178 (p = 0.225) for the ΔT1.

## Discussion

The main results of this study were as follows: RESOLVE yielded DWI of high quality in both small animals and KARs. In the small animal models, T1 and ADC values were correlated to IF and also to interstitial inflammation and could both efficiently discriminate diseased from healthy kidneys. In patients, adjusting absolute cortical T1 or ADC values to medullary ones by calculating the ΔT1 and ΔADC (difference between cortical and medullary T1 or ADC) improved IF assessment. ΔADC was negative in all allografts harboring more than 40% fibrosis and positive in allografts with less than 40% fibrosis. In KARs, ΔADC outperformed ΔT1 for IF detection.

In animal models, T1 significantly increased in diseased kidneys compared to controls. In contrast to the small animal models, only a moderate correlation between T1 and IF and no correlation between T1 and cellular inflammation parameters were observed in KARs. This discrepancy between small animal models and KARs was surprising and is not fully elucidated. T1 is sensitive to modification of kidney structure induced by fibrosis, but also to other factors such as inflammatory cell infiltration and mainly edema as previously described in more acute settings^42^,^43^. Major interstitial inflammation was not a preponderant finding in our KAR biopsies as attested by the Banff scores. Therefore, edema was likely more preeminent in the experimental models than in the more chronic situation of planned biopsies for allograft patients. This may explain the difference between the experimental models and the patients. As it remains very challenging to measure edema on histology, this hypothesis cannot directly be verified.

We observed a clear correlation between IF and ADC values, both in experimental models and in KARs. This was in agreement with previous studies that measured a reduced ADC *in vivo* in well-controlled animal models of fibrotic kidney compared to healthy kidneys^26^,^44^. Currently, 3 studies have investigated the relationship between renal IF and ADC^29^,^45^,^46^. In this first study, a lower ADC measured in the whole parenchyma was found in CKD patients compared to healthy volunteers^45^. However, ADC in the cortex and medulla was not evaluated separately in this study, as it was not possible to reliably discriminate both these kidney regions in CKD and healthy volunteers’ kidneys. In a second study, ADC correlated with allograft fibrosis, but not cell infiltration in delayed graft function patients at 1 week after transplant^46^. However, the extrapolation of their data to later times after transplantation (such as in our study) is not direct. The confounding effect of acute inflammation on this relationship is not yet well known, even if preliminary data suggested that it could be small. In the third study, Zhao *et al*. demonstrated a correlation between cortical ADC and IF in CKD^29^. Our present results are in agreement with these findings. Contrary to Zhao *et al*.^29^, who used absolute cortical and medullary ADC values, we introduced in this study a new index, ΔADC. This new index has several advantages to minimize the physiological inter-individual variation and optimize IF assessment in patients. A physiological variation in absolute ADC values was previously reported, even in healthy subjects, between individuals under different conditions of flow and tissue hydration^47^,^48^. After water loading, a significant and similar increase of the ADC of 7% in the cortex and 9% in the medulla was measured compared to the baseline^47^. Using ΔADC can minimize these causes of inter-individual variation as the intrinsic variation of ADC is corrected for by normalization from subtraction of the medullary ADC. In addition, the fibrosis changes affect preferentially the cortex. Although the medulla may also display kidney lesions in patients, we observed no correlation between medullary ADC and cortical fibrosis or eGFR in our patient population. This preferential localization of fibrosis also supports the efficiency of the ΔADC. Finally, normalization to the medulla was technically easier and more efficient than to surrounding tissues outside the kidney, since the close proximity of the medulla decreased errors related to B1 and Bo heterogeneity as well as to the coil sensitivity profile. We did not use ΔADC in the small animal models, as there was no large inter-individual variation of absolute cortical T1 or ADC observed. In addition the lack of separation of layers in the obstructed kidney made separate cortex and medulla ROI positioning impossible in the UUO model.

There are several limitations to the present study. Although we acquired 10 b values for the diffusion images, we did not use an IVIM model to fit the data. After preliminary testing, the fit of IVIM model was not sufficiently robust by comparison to the fit of the monoexponential model in agreement with previous observations^49^. We also decided to keep all 10 b values to improve the robustness of the monoexponential fit. The reduction of the number of acquired b values is certainly possible as shown recently in prostate diffusion^50^ and could be an efficient opportunity to reduce the acquisition time of the RESOLVE sequence. The optimal number of b values for a monoexponential fit in our clinical setting remains to be determined in a further study.

The size of our clinical cohort is relatively small and our population homogeneous. This homogeneity helps with validation in such a cohort, but restricts the knowledge on applicability in a wide range of pathologies. Our patients were KARs undergoing scheduled biopsies and acute pathologies were certainly under-represented. However, we were already able to observe a clear correlation in our sample, strengthening the value of RESOLVE in chronic lesions and specifically in IF evaluation. We distinguished patients with relatively ‘low level’ of fibrosis from patients with ‘high level’ of fibrosis across the threshold of 40%. As our population was not uniformly distributed along the linear regression line, we preferred to give accuracy and use the linear discriminant analysis technique instead of using areas under receiver operating characteristic curves. A larger size validation in more diverse, but separate, groups will therefore be needed in the future to generalize this observation to acute and other chronic pathologies, as well as to native kidney diseases. Other limitations include the fact that biopsy as a gold standard is subject to sampling bias whereas MR parameters were measured on multiple slices covering the entire kidney. Additionally, pathological methodology for evaluation of IF is still debated^38^. Finally, our experimental models may not be fully synonymous with KARs patients. Both the UUO and BSA nephritis models were chosen as they are classically used for experimental renal fibrosis, which was the parameter of interest in our study. It should also be emphasized that these models were used as a preliminary validation of the sensitivity of the RESOLVE sequence rather to reproduce chronic pathology expected in kidney transplant.

Although our observation is still preliminary, it already indicates that diffusion MRI with the RESOLVE sequence may specifically identify fibrosis extent in KARs and potentially, in the future, in other kidney patient populations. The correction of inter-individual variability of DWI by calculating the ΔADC will also render this method more reliable in the clinical setting. Although more work is needed before everyday clinical application, this tool will likely be valuable for the follow up of patients after therapeutic modifications and to assess the extent of chronic lesions in some patients where biopsy may not be recommended. Finally, this noninvasive method may give us a better assessment of renoprotective drug effects on structural aspects of the kidney, and not only on renal function and/or albuminuria.

In conclusion, we demonstrated that MRI can evaluate IF in experimental models and in kidney allograft recipients. Outperforming T1 mapping, diffusion MRI with the RESOLVE sequence allows differentiation of the cortex and medulla to measure the ΔADC, decreasing inter-patient variability and improving correlation to histopathological assessment of IF. Further studies in other types of CKD patients will be needed, but this new technique certainly responds to a need in the clinical setting.

## Methods

### Experimental animal models

All experiments were in strict accordance with the principles and guidelines of the Federal Veterinary Office for the Care and Use of Laboratory Animals and were approved by the Canton of Geneva animal experimentation ethics committee (1022/3898/2). All experimental procedures were done under Isoflurane inhalation anesthesia (1.5% O_2_ and air with 2–3% Isoflurane) and with monitoring during imaging using a respiratory pad (SA Instruments, Stony Brook, NY). Male Wistar rats were used for both models (Janvier, France, weighing 150–175g, aged two months at receipt). In the unilateral ureteral obstruction (UUO) model^51^, left ureters were visualized through a flank incision and double ligated with 6–0 silk. Animals were imaged and sacrificed at time points of 1 (n = 7), 2 (n = 6) or 3 (n = 3) weeks after ligation and tissue samples from obstructed and contralateral kidneys were collected for histology. The contralateral right kidney served as a control kidney in this model. A second model of interstitial inflammatory nephritis (IN) was induced using bovine serum albumin (BSA) injections in nephrectomized rats^37^. One week following left-sided nephrectomy, rats were randomly assigned to daily intraperitoneal injections of either 1g BSA in saline (Fraction V, No. A-4503, 96–99% albumin, Sigma Chemical Company, St. Louis, MO) (n = 6 with one deceased rat) or 0.9% saline alone (sham, n = 8). BSA animals were imaged and sacrificed at 3 weeks after the start of the injections. For both models, each time point consisted of separate groups of rats with a single MRI acquisition followed by immediate sacrifice and histologic assessment.

### Kidney Allograft Recipient

All subjects provided informed consent. The study was approved by the ethics committee at Geneva University Hospitals (CER 11–160) and conducted in accordance with the ethical guidelines set down in the Declaration of Helsinki (1975). The inclusion criteria for our study were patients undergoing a kidney biopsy scheduled for a clinical reason and absence of exclusion criteria. MRI was planned on the same day as the biopsy whenever possible and with a maximum of two weeks delay. Exclusion criteria were the presence of a pacemaker or other MR incompatible devices, pregnancy, claustrophobia, and refusal of patients. From August 2013 to June 2014, 90 KARs underwent scheduled kidney biopsy as part of their medical workup according to the kidney transplantation team at the University Hospital of Geneva. From these 90 patients undergoing biopsies, 40 KARs met the inclusion criteria and gave a written informed consent. Seven candidates were excluded as shown in the flow chart (Fig. 5). In the included patients, biopsy justifications were either systematic follow-up biopsy at 1, 5, 10 or 20 years after transplantation (n = 13), before stopping steroids (n = 3), or indication biopsies (n = 17). The reasons for indication biopsies were: apparition or rise of DSA (donor specific antibodies), suspicion of sub acute or chronic renal allograft rejection, subacute increase in serum creatinine levels above the baseline value, apparition of proteinuria or hematuria, control post rejection treatment and control post immunosuppressive therapy change. For practical reasons related to the availability of the MR systems, no patient undergoing emergency biopsy for suspicion of acute rejection was included in our study. Patient characteristics are described in Table 1.

### Histological fibrosis quantification

Both automated and visual analysis of histological fibrosis was performed. For automated quantification, histological slices with Sirius red staining were scanned on a Mirax 3DHistech microscope (20x objective, calibration 0.232 μm/pixel) and analyzed with Tissue Studio (version 3.60) software (Definiens AG, München, Germany, 2.1.0; Build 27594 × 64 version of Definiens Developer). For IF measurement, slides to be processed were assembled in workspaces for subsequent automatic analysis. Staining information (in general settings for processing) was selected as “IHC dual Brown/Red Chromogene”. The first step was selection of cortical area of the kidney where processing should be applied. This area was selected with manual region of interest (ROI) selection (Draw Polygons). Processing then used “Marker Area Detection” with the following parameters: “Threshold Hematoxylin” = 0.15, “Threshold Brown” = 0.59, “Threshold Red” = 0.11 and “Minimum area” = 10 μm^2^ for human sections. For Sirius red analysis in rats, the parameters were “Threshold Hematoxylin” = 0.07, “Threshold Marker” = 0.45 and “Minimum area” = 10 μm^2^. The selected polygonal ROIs were automatically processed on a Definiens server and results obtained from “Default Export” with all parameters. The parameters analyzed and reported in paper correspond to area of red marker area [μm] divided by total area of the selected ROI [μm], reported as a percentage. For the visual assessment of inflammation in experimental animals, sections were graded from 0 to 3 for inflammatory infiltrate, separately and in a blinded fashion by two experienced nephrologists. The mean value was then conserved. In patients, in addition to the automated analysis of the Sirius red staining, fibrosis and interstitial inflammation was assessed by an experienced clinical pathologist from the Masson trichrome and HE staining and graded in the Banff scoring system^40^,^41^ as well as a giving percentage for interstitial fibrosis. Histopathological changes including tubulitis (“t” score), interstitial inflammation (“i” score) and total interstitial inflammation (“ti” score) were added to define the tubule-interstitial inflammation Banff score (i+t+ti). Moreover, interstitial fibrosis (“ci” score) and tubular atrophy (“ct” score) were used to define the BANFF IF/TA (ci+ct) score. In one patient the ci and ct were not graded because the histological material was too small.

### MR imaging

MRI was carried out on a Siemens Magnetom Trio (Tim system) 3T clinical scanner (Siemens AG, Erlangen, Germany). Pseudo-coronal T1 maps were acquired with the Modified Look-Locker Inversion recovery (MOLLI) pulse sequence^17^. For DWI, both a conventional single-shot diffusion-weighted imaging sequence (ss-EPI) and ‘Readout Segmentation Of Long Variable Echo train’ (RESOLVE) sequence^52^ were acquired with the same resolution, shimming, GRAPPA factor and b-values. All parameters are given in Table 2. The optimized single-shot DWI with the same resolution and b values was attempted, but as the images were not of analyzable quality they are not reported in the table for simplicity.

### MRI image analysis

MRI image examinations were performed blinded to all clinical parameters and histologic results in each patient. In experimental animals, blinded analysis was not possible in the UUO model due to clear morphological differences, but was performed in the BSA model. MR Images were analyzed on an external workstation (OsiriX 5.5.2). The mean T1 or ADC was calculated as the mean of all pixels included in ROIs ± standard deviation from multiple ROIs. ADC was measured on quantitative ADC maps generated using a monoexponential model on a pixel-by-pixel basis.

#### Experimental animal models

A single ROI was placed exclusively in the renal cortex of all BSA animals and the control in the UUO model. The obstructed UUO kidney no longer showed a differentiation between the cortex and medulla. In this group, care was taken to avoid the dilated cavity and to remain in the solid part of kidney containing a mixture of cortex and medulla. Pearson’s correlations between MRI and histological parameters were carried out per group due to possible staining variations. Box plots and one-way analysis of variance (ANOVA) with post-hoc Bonferroni (SPSS 21.0) were used to assess statistical differences (p < 0.05 was statistically significant).

#### KARs

Multiple ROIs were placed in the cortex (n = 11 ± 3) and in the medulla (n = 19 ± 6) of the central and consecutive slices of each kidney. The SI of all these ROI was averaged to provide a single value for either the cortex or the medulla. Mean size of each individual ROI was 1.2cm^2^ ± 0.1 cm^2^ for the cortex with the range size 0.6–2.7 and 0.4 cm^2^ ± 0.04 cm^2^ for the medulla with the range size 0.2–0.7. To reduce T1 and ADC inter-individual variability in patients, indices ΔT1 and ΔADC were calculated as: ΔT1 = <T1_cortex_> − <T1_medulla_> and ΔADC = <ADC_cortex_> − <ADC_medulla_>. Correlations were considered significant when p < 0.05. Correlation coefficient comparison was performed using the Fisher Z-transform (http://www.fon.hum.uva.nl/Service/Statistics/Two_Correlations.html). Two observers also performed inter-observer agreement for the T1 and ADC values measured in the cortex and medulla, as well as ΔT1 and ΔADC. Ten KARs were chosen randomly and inter-observer reproducibility was calculated using Pearson’s correlations and Intra-class Correlation Coefficient (ICC) using one-way random single measures.

MRI and biopsy data were finally analyzed in order to define the best IF threshold detectable by DWI. IF was defined as a binary factor determining the presence or absence of fibrosis using thresholds from 10% to 70% in increments of 10%. After the IF percentage was transformed into a binary factor “high IF” or “low IF” (above or below a predefined fibrosis threshold), a non-parametric Wilcoxon test was used to compute the p-value between the both groups. The fibrosis threshold was selected at the level where the Wilcoxon test was the most significant. In a further analysis, a linear discriminant analysis allowing the classification of each ΔADC measure as normal or pathologic was performed to compute sensitivity and specificity of the DWI for the selected level of fibrosis defined previously by the Wilcoxon test. The accuracy was obtained using a bootstrapping method. Such resampling with 1000 bootstrap samples provided a nonparametric distribution of the accuracy and an estimation of the performance measure as a mean with confidence intervals (using software, R 3.1.1).

## Additional Information

**How to cite this article**: Friedli, I. *et al*. New Magnetic Resonance Imaging Index for Renal Fibrosis Assessment: A Comparison between Diffusion-Weighted Imaging and T1 Mapping with Histological Validation. *Sci. Rep.* **6**, 30088; doi: 10.1038/srep30088 (2016).

## Figures and Tables

**Table 1:** Characteristics of clinical and laboratory patient data.

	**All Patients (n** **=** **33)**	**Patients with RESOLVE Sequence (n** **=** **29)**
Clinical parameters		
Male (%)	69.7	69.0
Age (years)	54 +/−14	54 +/−14
Caucasian (%)	93.9	96.6
Deceased donor transplant (%)	63.6	65.5
Body Mass Index (kg/m2)	26.1 +/−4.9	26.0 +/−5.1
Systolic BP, mmHg	134.5 +/−21.1	134.6 +/−20.0
Diastolic BP, mmHg	81.5 +/−15.1	81.9 +/−15.2
Age of transplant (years)	9.8 +/−7.8	9.4 +/−7.5
Etiology of primary kidney disease (%)		
Diabetes	6.0	6.9
Hypertension	21.2	17.2
Glomerulonephritis	33.3	37.9
Polycystic kidney disease	15.2	17.3
Other	36.4	34.5
Comorbidities (%)		
Hypertension	84.9	82.8
Diabetes	18.2	17.2
Current smoker	12.1	10.3
Laboratory measurement		
Creatinemia (μmol/l)	180.2 +/−154.0	190.4 +/−161.8
eGFR (CKD-Epi, ml/min per 1.73m2)	50 +/−22	48 +/−23
Proteinuria (g/24h)	0.94 +/−1.8	1.02 +/−1.9
Albuminuria (%)		
<30 mg/g	46	48
30–300 mg/g	30	24
>300 mg/g	24	28
Immune suppression agents		
Steroids (%)	70	72
Calcineurin inhibitor (%)	85	86
Mycophenolate mofetil (%)	73	69
Sirolimus (%)	3	3
Biopsy – Histological lesions		
Fibrosis (%)		
Masson trichrome	28.2 +/−22.1	28.6 +/−23.5
Sirius Red automatized	25.8 +/−10.0	26.2 +/−10.4
BANFF score (mean and SD):		
i = interstitial inflammation	0.45 +/−0.33	0.52 +/−0.91
t = tubulitis	0.12 +/−0.33	0.14 +/−0.35
ti = total interstitial inflammation	0.45 +/−0.87	0.52 +/−0.91
g = glomerulitis	0.24 +/−0.61	0.24 +/−0.34
v = intimal arteritis	0.09 +/−0.38	0.10 +/−0,41
ptc = peritubular capillaritis	0.31 +/−0.64	0.36 +/−0.68
ci = interstitial fibrosis	1.44 +/−0.84	1.45 +/−0.87
ct = tubular atrophy	1.38 +/−0.90	1.38 +/−0.94
IF/TA (ci + ct)	2.81 +/−1.73	2.8 +/−1.80
Tubulo-interstitial inflammation (i + t + ti)	1.03 +/−1.94	1.17 +/−2.04

**Table 2:** MRI parameters for MOLLI T1 mapping and RESOLVE diffusion weighted imaging.

**MRI Sequence Parameters**	**MOLLI**	**RESOLVE**
Coil		
-In KARs	Phased-array abdominal & spine	Phased-array abdominal & spine
- In rats	Wrist	Wrist
TR/TE [ms]	711/1.09	2200/68
Acquisition time	1′5″	9′47″±4′
Resolution [mm^3^]		
- In KARs	2 × 2 × 5	2 × 2 × 5
- In rats	0.7 × 1.1 × 3.5	1.2 × 1.2 × 2.2
Echo spacing [ms]	2.6	0.32
Flip angle [°]	35	180
TI (inversion time) [ms]	161, 241, 321	No
Phase partial Fourier	6/8	Off
Number of shots per slice	No	5
GRAPPA factor	2	3
Number of signal averages	1	1
Gradients for b values > 0	No	3 orthogonal directions
b-values [s/mm^2^]	No	0, 10, 20, 40, 60, 150, 300, 500, 700, 900

Only the coils and the resolution were different between the experimental and clinical protocols.
